# Effects of chemical preconditioning on organic acid production from woody biomass

**DOI:** 10.3389/fchem.2026.1879689

**Published:** 2026-07-21

**Authors:** Sampath Gunukula, M. Clayton Wheeler, C. Luke Williams, David Thompson, Eliezer A. Reyes Molina, Ravikant Patil, Stephanie Ossai, Peter van Walsum, Xiaowen Chen

**Affiliations:** 1 Forest Bioproducts Research Institute and Chemical and Biomedical Engineering Department, University of Maine, Orono, ME, United States; 2 Idaho National Laboratory, Energy and Environment Science and Technology, Idaho Falls, ID, United States; 3 Department of Bioproducts and Biosystems Engineering, University of Minnesota, Minneapolis, MN, United States

**Keywords:** acid hydrolysis and dehydration, hemicellulose removal, levulinic acid, techno-economic analysis, woody biomass

## Abstract

**Introduction:**

Recovering hemicellulose from woody biomass prior to acid hydrolysis/dehydration (AHDH) processing can improve the yield and economics of levulinic acid (LA) production, but the effects of chemical preconditioning on subsequent LA yields and process economics remain to be characterized for softwood feedstocks.

**Methods:**

A pilot-scale chemical preconditioning system (CPS), operated with and without dilute sulfuric acid (0.25 wt%) and steam, was used to recover hemicellulose from raw white pine wood chips (39.5% glucan, 16.6% total hemicellulose [3.8% xylan, 10.1% mannan, 1.9% galactan, 0.7% arabinan], and 44.6% acid-insoluble lignin, oven-dry basis) prior to processing in a bench-scale AHDH reactor. Preconditioning was evaluated across a range of temperatures, residence times, biomass species, moisture contents, and particle sizes (1 mm to ∼18 mm) to determine effects on LA yield. A techno-economic analysis was also performed to estimate the minimum selling price of the resulting mixed sugar stream.

**Results:**

CPS effectively removed hemicellulose from wood chips, achieving complete xylan removal at 200 °C. Preconditioning at 170 °C for 30 minutes with 0.25 wt% H_2_SO_4_ increased LA yield from 0.21 mol/mol glucan (untreated) to 0.52–0.53 mol/mol glucan, a 2.5-fold improvement. CPS temperature, residence time, and biomass species influenced LA yields, whereas moisture content and particle size did not have a significant effect. The techno-economic analysis estimated a minimum selling price of $0.42/kg for a mixed sugar stream at ∼100 g/L concentration.

**Discussion:**

The LA yields achieved through mild chemical preconditioning (0.52–0.53 mol/mol glucan) were comparable to those obtained using more severe dilute acid (0.64 mol/mol) and SPORL (0.60 mol/mol) pretreatments, suggesting that CPS offers a less severe, potentially lower-cost alternative for enhancing LA production. The estimated minimum selling price of $0.42/kg falls within the $0.30–$0.60/kg range typical of commercial mixed C5/C6 fermentation feedstocks, indicating that CPS-based hemicellulose recovery is economically competitive and could be integrated into softwood-based AHDH processing without the need for more severe pretreatment conditions.

## 1 Introduction

The production of chemicals from non-renewable feedstocks (crude oil, natural gas, and coal) emits greenhouse gas (GHG) emissions that can lead to climate change impacts (Ma et al., 2023). The biochemical and chemical conversion technologies for upgrading lignocellulosic feedstock (e.g., woody biomass, corn stover, switchgrass and miscanthus, etc.) to bio-based chemicals and fuels have been developed as novel alternatives to the conventional petroleum-based derived ones. To catalyze the transition to bio-based chemical and fuel industry, platform chemicals have been identified (Werpy and Peterson, 2004). These platform chemicals are like fossil fuel derived building block chemicals that have potential to be upgraded to a wide range of end products. Levulinic acid is identified as a promising platform chemical with the potential for conversion into various chemicals and fuels (Werpy and Peterson, 2004). However, its use as a platform chemical hinges on economical production from lignocellulosic biomass, ideally achieving a cost of $1 per kilogram to make its industrial scale upgrading feasible (Gunukula et al., 2018).

Conventionally, levulinic acid can be produced from the acid hydrolysis and dehydration (AHDH) of lignocellulosic feedstock (Hayes et al., 2005). The AHDH process requires operating temperatures ranging from 180 °C to 200 °C with a dilute sulfuric acid concentration of 5 wt% and a residence time of 20–30 min, to attain optimal yields of levulinic acid (Hayes et al., 2005). These high temperatures, coupled with acid concentrations, pose safety and product contamination (e.g., material leaching) challenges, necessitating expensive reactor metallurgy to mitigate material leaching. To address this issue, lowering the AHDH process temperatures emerges as a potential solution to minimize reactor material concerns while maintaining efficient levulinic acid production. The pretreatment of lignocellulosic feedstock prior to the AHDH process may make it possible to attain a high levulinic acid yield at low reaction temperatures.

The carbohydrate content of woody biomass typically includes glucan, mannan, xylan, arabinan, and galactan (Jung and Oh, 2021; Marriott et al., 2016). Arabinan and galactan generally constitute less than 2% of the total oven-dry (OD) woody biomass by weight (Jung and Oh, 2021). Depending on the species of woody biomass, the glucan weight composition (on OD woody biomass basis) ranges from 38% to 45% by weight, mannan from 2% to 12%, and xylan from 4% to 18% (Ahmad et al., 2020). During the AHDH process, xylan typically gets converted to furfural. However, separation and purification of furfural from the hydrolysate containing furfural, levulinic acid, formic acid, sulfuric acid, soluble lignin, and other impurities (e.g., sugar derived by-products) using conventional solvent extraction is very complex and expensive (Mosier et al., 2005). Thus, cost effective recovery of xylan prior to the AHDH process can significantly reduce the overall costs of the AHDH process.

Dilute acid, sulfite, formic acid, and organosolv pretreatment of lignocellulosic feedstock have all been proposed to solubilize hemicellulose without affecting the native glucan structure prior to its conversion to fuels and chemicals to maximize biorefinery revenues and minimize the downstream processing issues (Chandel et al., 2014; Saha et al., 2005; Alvira et al., 2010; Zhu and Pan, 2010; Zhang et al., 2014; Qiao et al., 2023; Rabelo et al., 2023; Martin, 2021) Dilute acid pretreatment typically involves pretreating lignocellulosic feedstock using dilute acid (2 to 5 wt% H_2_SO_4_) at temperatures higher than 100 °C. Char formed within the reactor in the presence of dilute acid accumulates over time and sticks to the reactor walls, leading to practical issues with operating these processes continuously at industrial scale (Sluiter et al., 2012). The sticky nature of char could be attributed to the formation of adhesive materials from C5 sugar derived furfural and acid soluble lignin. Lowering the acid concentrations to below 1% during the dilute acid pretreatment may minimize the conversion of C5 sugars or furfural to resin type materials. Many of the problems associated with dilute acid pretreatment can be overcome with sulfite pretreatment of lignocellulosic biomass, formic acid pretreatment, and the organosolv process, as these methods solubilize C5 sugars and either solubilize lignin or modify its structure (Chandel et al., 2014). However, the commercial relevance of sulfite pretreatment is limited due to the formation of lignosulfonates and the lack of markets for them. Additionally, recovering C5 sugars from formic acid/organic solvent and formic acid/organic solvent for reuse is expensive, which hinders its commercial viability (Gunukula et al., 2021).

In this work, a combination of steam at temperatures between 170 °C to 200 °C, and dilute sulfuric acid (0 to 0.25 wt%), was applied to woody biomass as an alternative to pure steam or formic acid/Organosolv pretreatment for preprocessing woody biomass before conversion to organic acids using AHDH. Preprocessing included size reduction and steam/acid pretreatment performed at residence times from 15 min to 60 min in a small pilot-scale system in both batch and continuous modes for the pretreatment of clean white pine chips, loblolly pine chips and white pine residues. The efficacy of feedstock preconditioning was evaluated by quantifying the extent of hemicellulose removal from woody biomass, as well as measuring the yields of levulinic acid obtained from the preprocessed woody feedstock during the AHDH process. Finally, the techno-economic analysis (TEA) was conducted to assess the process economic impact of feedstock preconditioning using CPS unit. While hemicellulose pretreatment has been extensively studied in the context of enzymatic hydrolysis for bioethanol production, its systematic effect on downstream acid-catalyzed levulinic acid production has not been characterized at pilot scale using very dilute acid concentrations (≤0.25 wt%). This work addresses that gap by integrating pilot-scale CPS preconditioning with bench-scale AHDH conversion, spanning multiple feedstocks, particle sizes, moisture contents, temperatures, and residence times, and by assessing the economic viability of the preconditioning step through a targeted TEA. The principal novelty of this work is therefore threefold: (i) it is, to our knowledge, the first pilot-scale demonstration that hemicellulose removal sufficient to substantially improve downstream LA yield can be achieved with sulfuric acid loadings an order of magnitude below those used in conventional dilute-acid pretreatment (2–5 wt%) or SPORL (≥2 wt%); (ii) it links preconditioning severity (temperature, residence time, acid loading) directly to AHDH-stage LA yield across two woody feedstocks and both batch and continuous operating modes, rather than to enzymatic digestibility as in most prior hemicellulose-pretreatment studies; and (iii) it pairs this performance data with a process-scale techno-economic analysis of the resulting mixed sugar co-product stream. Dilute sulfuric acid was selected for the preconditioning step, rather than nitric, hydrochloric, or other mineral acids, because it is the acid already used industrially in the downstream AHDH process and in most reported lignocellulosic pretreatments, it is comparatively inexpensive and easy to recover or neutralize at scale, and prior dilute-acid pretreatment literature provided a well-characterized baseline against which the very low acid loadings used here could be benchmarked; a systematic comparison with other mineral and organic acids was outside the scope of the present pilot-scale study and is noted as a direction for future work.

## 2 Methodology

### 2.1 Feedstock

The raw, clean white wood pine and loblolly pine chips were initially size-reduced to ½ inch and ¾ inch using a G1635 Knife Mill (JRS – Birmingham, Alabama, United States). Subsequently, only the white wood pine was further milled to 1 mm using an M24P Crumbler (Forest Concepts – Auburn, Washington, United States) before being preconditioned in the CPS unit. To evaluate the impact of preconditioning and LA yields on forest residues, a third feedstock was prepared by blending the ½-inch clean loblolly pine chips with forest residues in a 50:50 weight ratio, following dust and dirt removal using a Spudnik air separator (Key Technologies – Walla, Washington, United States).

Initial moisture content (MC) after sizing for all materials was measured and ranged between 4.5-11.2 wt%. Then, MC for the sized materials was adjusted to 25 and 50 wt.% by estimating the additional amount of water needed to reach the desired moisture for the CPS run, the required water was added and mixed using a Colorado Mixer equipment, and all of them were stored on closed 35-gal plastic drums for 36 h until equilibrium was attained. Other feedstock samples were oven dried at 40 °C for 48 h to assess limited MC impact on the preconditioning treatment and LA yields.

For the acid preconditioning, the sized materials at their corresponding MCs were slightly impregnated with certain amount of acidic solution (1 wt.% H_2_SO_4_) to have a total acid concentration of 0.25 wt.% in the system. Meaning that the sample with highest MC requires the lowest amount of acidic solution to be added and *vice versa*. The acid impregnated samples were stirred and mixed homogeneously for about 30–60 min to ensure an even distribution and diffusion of the acid in the solids.

### 2.2 Chemical preconditioning system operating conditions

The CPS unit at INL operates at temperatures from ambient to 216 °C and pressures from ambient to 300 psig. The key operating parameters for each run are summarized in Table 1, including temperature, residence time, biomass loading (∼1.7 kg OD per vessel), acid concentration (0 or 0.25 wt% H_2_SO_4_), and liquid-to-solid ratio (2:1 v/w). The batch CPS unit consists of three parallel jacketed high-pressure vessels (A, B, and C), each loaded with the same feedstock under identical conditions; these three vessels therefore constitute experimental replicates for each run (Supplementary Figures S1, S2). Steam was supplied from the bottom of each vessel via a concentric weep-hole tube, with temperature and pressure monitored by thermocouples and pressure gauges at the top and bottom of each vessel. Once the target temperature was reached, conditions were held for the specified residence time, after which steam flow was stopped and the vessels were allowed to cool naturally. Solids and liquid samples were collected separately and stored frozen to prevent sugar degradation.

**TABLE 1:** Chemical preconditioning of white pine wood chips (softwood) at different conditions. Each run was conducted simultaneously in three parallel CPS vessels (A, B, and C) under identical conditions, which serve as experimental replicates. Reported solid yields are the mean of the three vessels.

Parameter	Run 1			Run 2			Run 3			Run 4		
Vessel used	Batch A	Batch B	Batch C	Batch A	Batch B	Batch C	Batch A	Batch B	Batch C	Batch A	Batch B	Batch C
Particle size reduced by	Knife mill	Knife mill	Knife mill	Knife mill	Knife mill	Knife mill	Crumbler	Knife mill	Knife mill	Knife mill	Knife mill	Knife mill
Particle size	1/2 inch	1/2 inch	1/2 inch	1/2 inch	1/2 inch	1/2 inch	1 mm	1/2 inch	3/4 inch	1/2 inch	1/2 inch	1/2 inch
MC (%)	0	25	50	50	50	50	50	50	50	50	50	50
Oven dry (OD): Acid sol	1 : 2	1 : 2	1 : 2	1 : 2	1 : 2	1 : 2	1 : 2	1 : 2	1 : 2	1 : 2	1 : 2	1 : 2
[Acid sol.] (0.25 vol%)	0.25	0.25	0.25	0.25	0.25	0.25	0.25	0.25	0.25	0.25	0.25	0.25
Temp (°C)	170	170	170	170	170	170	170	170	170	200	200	200
Time (min)	30	30	30	15	30	60	30	30	30	15	30	60
pH liquid	∼2	∼2	∼2	∼2	∼2	∼2	∼2	∼2	∼2	∼2	∼2	∼2
OD (kg)	1	1	1	1	1	1	1	1	1	1	1	1
Sample #	1	2	3	4	5	6	7	8	9	A	B	C
Solids wt. (kg)	1.7	1.7	1.7	1.7	1.7	1.7	1.7	1.7	1.7	1.7	1.7	1.7
Liquid vol. (L)	∼2.5	∼2.5	∼2.5	∼2.5	∼2.5	∼2.5	∼2.5	∼2.5	∼2.5	∼2.5	∼2.5	∼2.5
Yield (g preconditioned solids per g-OD)	0.88	0.89	0.87	0.87	0.87	0.92	0.92	0.88	0.89	0.92	0.91	0.92
Sample#	1	2	3	4	5	6	7	8	9	10	11	12

### 2.3 Characterizing the raw and CPS pre-processed wood

The National Renewable Energy Laboratory’s standard procedure called Determination of Structural Carbohydrates and Lignin in Biomass, Laboratory Analytical Procedure (LAP) (Sluiter et al., 2012) was used to determine the composition of raw woody biomass and pretreated/preconditioned biomass samples. A summary of the procedure is as follows: All samples were dried in a fume hood to reduce their moisture content to less than 10% and stored in zip-lock bags to maintain the moisture content. The dried samples were then ground to about 0.5 mm particle size using a willy mill. Wood samples were extracted using acetone (Soxhlet Extraction) prior to carbohydrate analysis to remove extractives that could interfere with the downstream analytical steps. All samples were analyzed in duplicate. About 150 mg (oven-dry basis) of each sample was used for the analysis. All the samples along with two to three Sugar Recovery Standards (SRS) were hydrolyzed and then neutralized using Calcium Carbonate following the NREL LAP procedure. The neutralized hydrolysate and SRS samples and calibration solutions were analyzed for monosugars using a High-Performance Liquid Chromatography (HPLC, Shimadzu Scientific Instruments, Inc., Columbia, MD, United States) equipped with a Refractive Index Detector (RID) and Bio-RAD Aminex HPX-87P column with appropriate guard columns. The injection volume and column temperature were 25 μL and 80 °C, respectively. It should be noted that no attempt was made to correct the concentration of hemicelluloses for minor components such as uronic anhydride and acetic acid. Based on this procedure, the raw (untreated) white pine wood chips used as the clean feedstock (Table 2, sample 0) were composed of 39.5% glucan, 3.8% xylan, 10.1% mannan, 1.9% galactan, and 0.7% arabinan (total hemicellulose of 16.6%) and 44.6% acid-insoluble lignin on an oven-dry weight basis; the corresponding composition for the clean debarked loblolly pine whole-tree chips (Table 3) was 38.8% glucan, 8.2% xylan, 7.9% mannan, 2.8% galactan, and 1.9% arabinan (total hemicellulose of 20.8%).

**TABLE 2:** Carbohydrate analysis of chemical preconditioned biomass (white pine) solids and organic acid yields.

Sample	Glucan	Xylan	Galactan	Arabinan	Mannan	Acid insoluble lignin (wt%)	Fa yield based on glucan (mol/mol)	LA yield based on glucan (mol/mol)
0 (Clean)	39.52%	3.82%	1.94%	0.73%	10.12%	44.58%	0.32	0.21
1	37.60%	1.80%	0.45%	0.20%	5.30%	56.10%	0.42	0.52
2	43.80%	2.40%	0.75%	0.29%	6.87%	46.70%	0.35	0.42
3	44.30%	1.00%	0.56%	0.21%	5.42%	49.50%	0.42	0.53
4	45.60%	1.70%	0.50%	0.20%	5.20%	47.50%	0.35	0.46
5	44.60%	1.30%	0.20%	0.13%	3.91%	50.60%	0.39	0.47
6	44.70%	1.10%	0.21%	0.11%	1.10%	51.20%	0.42	0.46
7	43.70%	0.90%	0.17%	0.08%	2.32%	53.70%	0.49	0.53
8	45.40%	1.10%	0.21%	0.10%	3.26%	46.10%	0.46	0.46
9	43.70%	1.20%	0.18%	0.10%	2.95%	38.80%	0.39	0.41
10	45.75%	0.00%	0	0	0.61%	53.70%	0.39	0.24
11	36.13%	0.00%	0	0	0.72%	63.20%	0.39	0.27
12	42.41%	0.00%	0	0	0.60%	57.00%	0.35	0.2

Representative HPLC, chromatograms were included in the Supplementary Material (Supplementary Figure S3).

**TABLE 3:** Carbohydrate analysis of chemical preconditioned biomass (loblolly pine with and without residues) solids and organic acid yields.

Sample name	Glucan %	Xylan %	Galactan %	Arabinan %	Mannan %	Total C6%	LA yield (mol/mol C6)	Fa yield (mol/mol C6)
Clean debarked whole tree chips bulk	38.8	8.2	2.8	1.9	7.9	49.5	0.35 ± 0.06	0.42 ± 0.04
Residue blend; CPS; 185C; 0.25 wt% H_2_SO_4_	35	1.6	1.2	0.2	1.6	37.8	0.49 ± 0.013	0.35 ± 0.035
Residue blend; CPS; 170C; H2O	32.3	5.5	2.6	0.8	5.5	40.4	0.42 ± 0.06	0.31 ± 0.03
Residue blend; CPS; 170C; 0.25 wt% H_2_SO_4_	37.6	3.7	1.8	0.7	3.4	42.9	0.49 ± 0.06	0.35 ± 0.03
Residue blend; CPS; 200C; H2O	36.1	2.7	0.8	0.3	1.9	38.9	0.50 ± 0.06	0.35 ± 0.088
Residue blend; CPS; 185C; H2O	34.3	3.8	1.6	0.5	3.6	39.6	0.45 ± 0.04	0.32 ± 0.02
Residue blend; CPS; 200C; H_2_SO_4_	38.3	1.5	0.8	0.3	1.1	40.1	0.54 ± 0.01	0.35 ± 0.035
Clean tree; CPS; 170C; H_2_SO_4_	47.4	3.7	1.3	0.7	3.3	52	0.56 ± 0.06	0.37 ± 0.04
Clean tree; CPS; 185C; H2O	40.4	6	2.2	0.7	7.5	50.1	0.46 ± 0.06	0.33 ± 0.035
Clean tree; CPS; 185C; H_2_SO_4_	47	1.4	0.8	0.2	1.5	49.2	0.46 ± 0.013	0.37 ± 0.035
Clean tree; CPS; 170C; H2O	40.8	6.2	2.3	1	7.5	50.5	0.54 ± 0.06	0.46 ± 0.04
Clean tree; CPS; 200C; H2O	41.7	4.4	1.4	0.4	4.2	47.4	0.45 ± 0.03	0.35 ± 0.035
Clean tree; CPS; 200C; H_2_SO_4_	46	1	0.7	0.3	1.1	47.7	0.57 ± 0.06	0.39 ± 0.04
1/2″ knife mill, 180C-20 min, water, washed and dried (continuous)	47.2	2.7	1.1	0.4	6.6	54.9	0.36 ± 0.02	0.39 ± 0.035
1/2″ knife mill, 180C-20 min, H_2_SO_4_ (continuous)	42.8	2.7	1.1	0.6	8.2	52.2	0.50 ± 0.02	0.46 ± 0.04

### 2.4 Characterizing the CPS hydrolysates

The CPS hydrolysate was analyzed for C-5 and C-6 sugars, organic acids, sulfuric acid, 5-Hydroxymethylfurfural (HMF), and furfural using Shimadzu HPLC equipped with a RID and Bio-RAD Aminex HPX-87H column. The column temperature was 60 °C and 0.5 mM sulfuric acid was used as the eluent at the flow rate of 0.6 mL/min. The HPLC was calibrated using reagent-grade chemicals. The hydrolysates were analyzed before and after post-hydrolysis to determine the relative composition of oligomeric and monomeric sugars. Unless otherwise stated, all sugar concentrations reported in Tables 4, 5 represent total sugars after post-hydrolysis.

**TABLE 4:** Characterization of hydrolysates produced from the preprocessing of white pine wood chips. All sugar concentrations represent total sugars after post-hydrolysis.

Sample	Arabinose (g/L)	Glucose (g/L)	Xmg (g/L)	Formic acid (g/L)	Levulinic acid (g/L)	HMF (g/L)	Furfural (g/L)
1	1.563	6.984	19.586	0.988	1.766	1.640	0.390
2	2.444	7.176	24.999	1.043	1.806	1.745	0.322
3	2.347	7.556	23.897	1.237	1.845	2.041	0.344
4	1.583	7.841	19.091	0.989	1.747	1.712	0.281
5	1.253	7.063	15.282	0.936	1.718	1.850	0.377
6	0.615	4.365	7.807	0.619	0.604	1.584	0.444
7	1.067	7.517	10.641	0.996	1.111	2.402	0.547
8	1.66	6.357	19.519	0.778	0.922	1.827	0.294
9	1.132	8.488	13.711	1.059	1.159	2.443	0.435
10	2.986	0.930	0.432	0.731	0.733	1.432	0.065
11	12.631	2.540	0.184	2.160	3.773	3.649	0.596
12	1.795	0.748	0.248	0.861	0.548	1.245	0.722

**TABLE 5:** Characterization of hydrolysates produced from the preprocessing of loblolly pine wood chips. All sugar concentrations represent total sugars after post-hydrolysis.

Sample	Temp. (°C)	Acid content (wt%)	Glucose (g/L)	Xmg (g/L)	Arabinose (g/L)	Formic acid (g/L)	Levulinic acid (g/L)	HMF (g/L)	Furfural (g/L)
CT^a^	170	0.25	3.224	14.955	1.765	0.758	0.881	0.687	0.194
RB^b^	170	0.25	6.820	27.828	3.889	1.480	1.642	1.434	0.428
CT	170	0	0.026	3.913	5.865	0.430	0.262	0.260	0.000
RB	170	0	0.995	9.682	3.913	1.404	0.886	0.409	0.000
CT	185	0.25	5.783	7.021	0.910	1.006	0.569	1.962	0.712
RB	185	0.25	7.116	16.460	0.000	0.512	0.733	3.268	1.102
CT	185	0	0.872	9.146	2.301	1.324	1.015	0.767	0.335
RB	185	0	1.981	13.216	2.642	1.827	1.616	1.616	0.929
RB	200	0.25	5.918	10.722	0.000	0.639	0.958	3.166	3.927
CT	200	0.25	5.540	5.366	0.700	1.394	0.649	1.691	2.447
CT	200	0	2.303	5.484	0.000	2.205	2.052	2.353	1.501
RB	200	0	1.548	2.276	0.000	2.040	1.662	1.534	0.899

^a^ clean tree.

^b^ whole tree including bark.

### 2.5 Determination of insoluble and soluble lignin in CPS hydrolysates

1.5 mL of 72% sulfuric acid (H2SO4) was added to 42 mL of each liquid CPS hydrolysate sample. Following acidification, the mixture was hydrolyzed in an autoclave at 121 °C for 1 h. Once hydrolysis was complete, deionized water was added to bring the sample to its initial volume, and the acid-insoluble lignin was separated from its soluble constituents using filtering crucibles, followed by a 24 h drying at 120 °C. Acid-soluble lignin from the filtrates was determined from the absorbance at a wavelength of 205 nm using the Shimadzu UV Spectrophotometer.

### 2.6 Bench scale AHDH operating conditions and characterizing AHDH hydrolysates

A Parr glass reactor with a volume of 1 L with an agitator was used to perform bench-scale AHDH experiments. The preconditioned biomass solids were combined with a 5 wt% sulfuric acid solution at a solid-to-liquid ratio of 1:10 (w/v) prior to reaction. The AHDH reactor was intentionally operated at 140 °C–150 °C — below the typical pilot-scale AHDH temperature of 180 °C–200 °C—to isolate the effect of CPS preconditioning on LA yield under milder conversion conditions and to remain within the safe operating range of the glass Parr reactor. At this temperature, the pressure in the reactor reached between 4.5 and 5.5 bar. The residence time at reaction temperature was 60 min. The reactor contents were heated using heating oil to the desired temperature at a heating rate of 1.1 °C per minute. The reactor contents were cooled to room temperature using cold water at 1.1 °C per minute. The AHDH hydrolysate is filtered using a vacuum filter to separate and collect char. The solid-free AHDH hydrolysate was analyzed using HPLC (a Shimazdu HPLC system equipped with a RID detector and BioRAD Aminex HPX-87H column (similar to Section 2.4)) to determine the formic and levulinic acid yields.

## 3 Results and discussion

### 3.1 Preprocessing of white pine wood chips using the batch chemical preconditioning system

The feedstock preconditioning variables investigated with preprocessing white pine chips include particle size, residence time, temperature (170 °C and 200 °C), and initial moisture content (Table 1). Preconditioned solid yields ranges from 0.87 g/g to 0.92 g/g (Table 1). It is expected that the preconditioned solid yields reduce as the temperature and the residence time increases. However, we did not notice such a trend, which could be attributed to the formation of char from sugar derived products like furfural, hydroxymethyl furfural, levulinic acid, and formic acid, especially at high temperature of 200 °C. The chemical composition of preprocessed hydrolysates of white pine chips at different conditions are presented in Table 4. The chemical compositional analysis of extracts (hydrolysates) of white pine wood chips has shown that these extracts contain C-5 and C-6 sugars, acid soluble lignin, acid insoluble lignin, and sugar derivatives (formic acid, furfural, hydroxy methyl furfural, and levulinic acid). The hemicellulose (xylan, mannan, arabinan, and galactan) removal from white pine wood chips increase with preconditioning temperature, and can be completely removed at 200 °C.

Preconditioned biomass solids were subjected to the AHDH process, and the measured yields of organic acids (levulinic acid and formic acid) are presented in Table 2. The results indicate that, when compared to unprocessed or clean white pine wood chips (sample 0 in Table 2), the yields of organic acids from the preconditioned white pine sawdust are significantly higher. This increase in yields is hypothesized to result from increased cellulose accessibility in the biomass solids following preconditioning, consistent with the removal of hemicellulose components such as xylan, galactan, and mannan observed in Table 2. Structural characterization (e.g., SEM, BET surface area) to directly confirm changes in porosity is planned for future work.

Preconditioning temperature and residence time are found to influence the yields of organic acids from white pine sawdust (Tables 1, 2). Of the tested conditions, a residence time of 30 min at 170 °C in a batch system attained the highest yields of levulinic and formic acid during the AHDH process (Tables 1, 2). Increasing temperature to 200 °C greatly reduced the yields of levulinic acid (Samples 10, 11, and 12 in Table 2), with LA yields dropping to 0.20–0.27 mol/mol glucan compared to 0.46–0.53 mol/mol at 170 °C. Notably, formic acid yields remained comparable between the two temperatures, suggesting LA-specific degradation rather than a general loss of conversion efficiency. This behavior is consistent with secondary degradation pathways well established in the literature: at elevated temperatures and under acidic conditions, LA can undergo further degradation, while HMF and furfural are known to polymerize and repolymerize with acid-soluble lignin fragments to form insoluble humin/char deposits. The observation that solid yields did not decrease monotonically with temperature (Table 1) supports this interpretation, as char deposition on the biomass surface would offset mass loss from hemicellulose solubilization. Direct quantification of char yield was not performed in this study and is identified as a priority for future work.

Interestingly, the initial moisture content of pine during conditioning did not significantly affect the yields of levulinic acid and formic acid (Samples 1, 2 and 3 in Tables 1, 2). Drying biomass, which leads to the collapse of the porous structure, did not lead to different results at this temperature and acid loading during preconditioning. Additionally, organic acid yield results show that reducing the particle size beyond <1/2 inch by over a factor of ten did not have a significant impact on the yields of levulinic acid and formic acid (Samples 3 and Sample 7 in the Table 2). Together, these results indicate that these pretreatment conditions are severe enough that they can handle a wide range of preprocessing heterogeneity.

Further exploration of preprocessing operations was accomplished by comparing the CPS preconditioning to dilute acid pretreatment at elevated sulfuric acid concentrations and the sulfite pretreatment SPORL process. The National Renewable Energy Laboratory provided dilute acid and SPORL preprocessed white pine solids for the AHDH process. For the dilute acid pretreatment method, 1.5 kg of white pine wood chips were submerged in a solution comprising 15 L containing 2% sulfuric acid and allowed to soak overnight. Following this, the soaked wood chips underwent dewatering via a small Vincent screw press until the moisture content reached 30% solids. Approximately 1 kg of the dewatered biomass was then loaded into a two gallon Parr reactor. Utilizing direct steam injection, the temperature of the biomass was elevated to 200 °C. Sustaining this temperature for 10 min, the biomass was subsequently cooled overnight. For the SPORL process, 1 kg of white pine underwent pretreatment using a solution composed of 9% sodium bisulfite and 2.2% sulfuric acid, with a solid-to-liquid ratio of 1:4. The biomass was subjected to a ramp-up process, incrementally raising the temperature to 165 °C over 75 min. Upon reaching the desired temperature, the biomass was maintained at 165 °C for 55 min. The yields of organic acids from the dilute acid and SPORL preprocessed pine solids during the AHDH processes were listed in Table 6. The comparison of organic acid yields in Table 6 to that of Table 2 suggests that the CPS process, using only 0.25 wt% sulfuric acid, achieves LA yields comparable to those obtained with higher acid loadings and the SPORL pretreatment process, while being considerably less severe and more practically feasible at industrial scale (Table 7).

**TABLE 6:** Compositional analysis of SPORL and Dilute acid preprocessed solids and the organic acid yields under acid hydrolysis and dehydration conditions.

Sample #	Sample name	Glucan %	Xylan %	Levulinic acid yield (mole LA/mole glucan)	Formic acid yield (mole FA/mole glucan)
1	SPORL	48.07	0	0.60 ± 0.03	0.28 ± 0.04
2	Dilute acid	46.21	0.34	0.64 ± 0.06	0.35 ± 0.03

**TABLE 7:** Comparative summary of preprocessing severity and levulinic acid (LA) yield for untreated biomass, CPS preconditioning (this work), and literature dilute-acid/SPORL pretreatments. CPS achieves LA yields comparable to substantially harsher acid pretreatments while using an order-of-magnitude lower acid loading.

Pretreatment	Acid loading	Temp (°C)	Time (min)	LA yield (mol/mol glucan)	Source/Note
Untreated white pine chips (baseline)	0	—	—	0.21	Table 3, sample 0
CPS preconditioning, white pine (this work, optimum)	0.25 wt% H_2_SO_4_	170	30	0.52–0.53	Table 3, samples 3 and 7
CPS preconditioning, loblolly clean tree (this work)	0.25 wt% H_2_SO_4_	200	30	0.57 ± 0.06	Table 6
CPS preconditioning, continuous mode (this work)	0.25 wt% H_2_SO_4_	180	20	0.50 ± 0.02	Table 6
Dilute acid pretreatment, high concentration (literature)	2 wt% H_2_SO_4_	200	10	0.64 ± 0.06	Table 4
SPORL pretreatment (literature)	9% NaHSO_3_ + 2.2% H_2_SO_4_	165	55	0.60 ± 0.03	Table 4

### 3.2 Preprocessing of loblolly pine wood chips using batch and continuous chemical preconditioning system

We analyzed the effectiveness of dilute acid pretreatment on clean debarked loblolly pine wood chips and a residue blend of loblolly pine wood chips. The batch dilute acid and stream treatment was done at three different temperatures (170 °C, 185 °C, and 200 °C), a residence time of 30 min, and with either pure steam or an additional 0.25 wt% sulfuric acid solution. It was observed that the addition of a 0.25 wt% sulfuric acid solution increased the removal of hemicelluloses (Tables 3, 5). Interestingly, the hydrolysate composition of preprocessed loblolly pine chips indicates the presence of residues led to an increase in hemicellulose removal from the loblolly pine chips as well as increase in the conversion of cellulose to organic acids (Tables 3, 5). This could be because naturally occurring organic acids present in the residues may have slightly lowered the pH of the CPS hydrolysate, thereby increasing the effective acid concentration during preconditioning. The measured pH values for the clean tree and residue blend CPS runs at all temperatures have been added to Table 5 to support this interpretation. Unlike preprocessed white pine solids, the galactan and mannan content of preprocessed loblolly pine solids are found to be significantly higher and the levulinic acid yields of preprocessed loblolly pine residues increase with the temperature and were the highest at 200 °C (Table 3). Future studies will concentrate on detailed surface characterization of dilute acid treated white pine chips and loblolly pine chips using scanning electron microscopy to gain insight into the lower organic acid yields observed with white pine preprocessed at 200 °C.

In addition to the batch dilute sulfuric acid and steam treatments, continuous steam and acid treatment tests on larger wood chips were performed. The continuous CPS process at 180 °C and residence time of 20 min is used to preprocess loblolly pine chips with water and acid solution with 0.25 wt% sulfuric acid. One notable advantage of the continuous process is its potential scalability for industrial implementation. By continuously feeding wood chips into the system and maintaining a steady temperature and residence time, manufacturers can achieve consistent results and higher throughput compared to batch processing methods. The levulinic acid yields from the continuous process with sulfuric acid are comparable to those of batch operations (Table 3), despite the shorter total acid contact time and reduced time spent on heating and cooling in the continuous mode. It is important to note that the batch and continuous CPS runs differ not only in residence time but also in thermal history: the batch process involves a more gradual heat-up and extended cool-down, whereas the continuous screw-conveyor system reaches the target temperature more rapidly and has a narrower residence time distribution. These differences in acid contact time and thermal exposure make direct comparison of batch and continuous severity non-trivial. Nevertheless, the comparable LA yields suggest that the continuous process achieves effective preconditioning within its shorter operational window. Interestingly, the continuous process yielded higher formic acid compared to batch, which may reflect differences in cellulose accessibility or organic acid degradation kinetics under the two operational modes.

### 3.3 Techno-economic assessment of the preconditioning process

The TEA of the AHDH process to produce furfural, levulinic acid, and formic acid in a biorefinery plant with a feedstock processing capacity of 2000 metric ton per day was published by Gunukula (Martin, 2021) In this TEA model, the feedstock (white pine sawdust) cost was assumed at $88 per dry metric ton at the biorefinery factory gate. The addition of preconditioning step in the biorefinery prior to the AHDH process can increase the feedstock cost at the AHDH reactor throat. However, the recovery of xylan and mannan prior to AHDH process and selling this sugar stream at a concentration of 100 g/L could generate an additional income to the biorefinery and may offset the additional costs associated with the preconditioning step. The capital costs of adding preconditioning reactor, flash column, and condenser AHDH biorefinery were assessed at $34 MM. The annual operating costs for recovering mixed sugar stream at a concentration of about 100 g/L were calculated at $37 MM. Here, the feedstock cost ($88 per metric ton) was allocated to cellulose, hemicellulose, and lignin depending on the chemical composition (Table 4, sample 0 or clean). Other TEA assumptions were the same as our published study. The minimum selling price of the mixed sugar stream, with a sugar concentration of approximately 100 g/L, produced from preconditioning white pine sawdust at 170 °C with a residence time of 30 min, was found to be $0.42 per kg. This result indicates that producing a mixed sugar stream for further upgrading via fermentation is cost competitive. For context, mixed C5/C6 hemicellulosic sugar streams are typically valued in the range of $0.30–$0.60/kg as fermentation feedstocks, placing the CPS-derived stream within a commercially relevant range. The TEA presented here is scoped specifically to the CPS sugar stream and should be read alongside our previously published full biorefinery TEA (Gunukula et al., 2021), which addresses downstream upgrading costs and sensitivity to feedstock price and yield variation.

### 3.4 Significance and outcome of this study

The combined preconditioning/AHDH/TEA results presented here indicate that very dilute sulfuric acid (0.25 wt%) preconditioning is an effective and economically relevant strategy for de-risking levulinic acid production from woody biomass. Practically, the key outcome is that hemicellulose removal sufficient to roughly 2.5-fold increase downstream LA yield (0.21 to 0.52–0.53 mol/mol glucan) can be obtained at an acid loading 8- to 20-fold lower than conventional dilute-acid (2–5 wt%) or SPORL pretreatments, while avoiding the lignosulfonate by-product and solvent-recovery costs associated with sulfite and organosolv methods, respectively (Table 7). This has two direct implications for biorefinery design. First, because acid loading, reactor metallurgy requirements, and neutralization/waste-handling costs scale with acid concentration, the low-severity CPS conditions identified here should reduce both capital and operating costs relative to harsher pretreatments, even though this was not separately quantified in the present TEA and is recommended as a follow-on techno-economic comparison. Second, the recovered xylan/mannan-rich sugar stream itself has standalone commercial value: at a modeled minimum selling price of $0.42/kg, it falls within the $0.30–$0.60/kg range reported for mixed C5/C6 fermentation feedstocks, so the preconditioning step can be operated as a co-product-generating unit rather than a pure cost center. The data also show that preconditioning effectiveness is governed primarily by temperature and residence time rather than by feedstock particle size or moisture content (Tables 1–3), and that hydrolysate pH, which fell in the ∼2 range for all acid-catalyzed runs (Table 1) and decreased further when naturally occurring organic acids from residue blends were present (Table 5), correlates with the extent of hemicellulose removal and the resulting LA yield. This pH dependence suggests that the residence time and acid concentration could, in principle, be traded off against one another within the narrow severity window explored here (15–60 min; 0–0.25 wt% H_2_SO_4_) to further tune the process for a given feedstock, which is a practical lever for plant operators managing variable incoming biomass quality.

## 4 Conclusion and future direction

To maximize the economics of the AHDH biorefinery, xylan needs to be recovered prior to the AHDH conversion as it causes downstream challenges. Preprocessing white pine chips with very dilute acid (0.25 wt% H_2_SO_4_) and steam at 170 °C for 30 min was found to effectively recover xylan and mannan. This preconditioning enhanced LA yields from 0.21 mol/mol glucan for untreated chips to 0.52–0.53 mol/mol glucan for preconditioned solids — a 2.5-fold improvement — comparable to more severe dilute acid (0.64 mol/mol) and SPORL (0.60 mol/mol) pretreatment processes. At acid loadings of 0.25% the initial moisture content of the chips, and particle sizes ranging from 1 mm to 12 mm did not have a significant impact on the efficacy of the preconditioning. However, the residence time and temperature were found to influence its effectiveness. The optimal temperature and residence time of the preconditioning step to achieve high yields of organic acids via the AHDH process are found to be dependent on the biomass species. This result highlights the importance of feedstock quality control and being able to change process conditions as needed to match changing feedstocks.

HPLC analysis of all the preconditioned hydrolysates in this work indicates the presence of C-5 and C-6 sugars in addition to the sugar derived products like HMF, furfural, and organic acids. The TEA analysis indicates that it requires about $0.42 to produce this mixed sugar stream at a concentration of about 100 g/L. The valorization of this hydrolysate stream is crucial for achieving economic viability of the AHDH biorefinery. Future work needs to be focused on growing different oleaginous yeast strains on CPS hydrolysates to determine the optimal microbial strain for accumulating fats. The optimal yeast strain will need to be studied as a potential feedstock for making transportation fuels via hydrothermal liquefaction technology. Looking ahead, three directions are identified as priorities for advancing this work toward industrial implementation. First, direct structural and chemical characterization (e.g., SEM imaging, BET surface area analysis, and FT-IR spectroscopy of solids before and after preconditioning and after organic acid separation) is needed to confirm the porosity, cellulose-accessibility, and functional-group changes that are currently inferred indirectly from compositional and yield data. Second, a broader screening of preconditioning acids (e.g., nitric, hydrochloric) and of acid concentration/temperature/residence-time combinations beyond the ranges tested here would clarify whether sulfuric acid remains the optimal choice at this very low severity and would help define the full operating envelope for the CPS unit. Third, continuous-mode operation should be scaled and run for extended periods to confirm that the char-handling and throughput advantages suggested by this pilot-scale work translate to sustained industrial operation, and the preconditioning step should be incorporated explicitly into an updated techno-economic and life-cycle assessment of the full AHDH biorefinery alongside valorization of the recovered sugar stream.

## Data Availability

The original contributions presented in the study are included in the article/Supplementary Material, further inquiries can be directed to the corresponding author.

## Author disclaimer

The views and opinions of the authors expressed herein do not necessarily state or reflect those of the United States Government or any agency thereof. Neither the United States Government nor any agency thereof, nor any of their employees, makes any warranty, expressed or implied, or assumes any legal liability or responsibility for the accuracy, completeness, or usefulness of any information, apparatus, product, or process disclosed, or represents that its use would not infringe privately owned rights.

## References

[B1] Ahmad Z. Al Dajani W. W. Paleologou M. Xu C. (2020). Sustainable process for the depolymerization/oxidation of softwood and Hardwood kraft lignins using hydrogen peroxide under ambient conditions. Molecules 25 (10), 2329. 10.3390/molecules25102329 32429419 PMC7287583

[B2] Alvira P. Tomás-Pejó E. Ballesteros M. Negro M. J. (2010). Pretreatment technologies for an efficient bioethanol production process based on enzymatic hydrolysis: a review. Bioresour. Technol. 101 (13), 4851–4861. 10.1016/j.biortech.2009.11.093 20042329

[B3] Chandel A. K. Antunes F. A. Anjos V. Bell M. J. Rodrigues L. N. Polikarpov I. (2014). Multi-scale structural and chemical analysis of sugarcane bagasse in the process of sequential acid–base pretreatment and ethanol production by Scheffersomyces shehatae and Saccharomyces cerevisiae. Biotechnol. Biofuels 7, 63. 10.1186/1754-6834-7-63 24739736 PMC4005856

[B4] Gunukula S. Klein S. J. W. Pendse H. P. DeSisto W. J. Wheeler M. C. (2018). Techno-economic analysis of thermal deoxygenation based biorefineries for the coproduction of fuels and chemicals. Appl. Energy 214, 16–23. 10.1016/j.apenergy.2018.01.065

[B5] Gunukula S. Schwartz T. J. Pendse H. DeSisto W. J. Wheeler M. C. (2021). Assessing the economic viability of pretreatment technologies to make sugars for chemical catalytic upgrading to fuels and chemicals. Sustain. Energy Fuels 5, 5513–5522. 10.1039/d1se01097b

[B6] Hayes D. J. Fitzpatrick S. Hayes M. H. B. Ross J. R. H. (2005). “The biofine process – production of levulinic acid, furfural, and formic acid from lignocellulosic feedstocks,” in Biorefineries: Industrial Processes and Products, Chapter 7 (Wiley). 10.1002/9783527619849.ch7

[B7] Jung H. J. Oh K. K. (2021). Production of bio-based chemicals, acetic acid and furfural, through low-acid hydrothermal fractionation of pine wood (Pinus densiflora) and combustion characteristics of the residual solid fuel. Appl. Sci. 11 (15), 7435. 10.3390/app11167435

[B8] Ma S. Lei T. Meng J. Liang X. Guan D. (2023). Global oil refining's contribution to greenhouse gas emissions from 2000 to 2021. Innovation 4 (1), 100361. 10.1016/j.xinn.2022.100361 36594044 PMC9804246

[B9] Marriott P. E. Gómez L. D. McQueen-Mason S. J. (2016). Unlocking the potential of lignocellulosic biomass through plant science. New Phytol. 209 (4), 1366–1381. 10.1111/nph.13655 26443261

[B10] Martin J. (2021). “Project liberty: launch of an integrated bio-refinery with eco-sustainable and renewable technologies,” in Conversion of Corn Stover Biomass to Bio-Ethanol, Final Report. Washington DC, United States: OSTI.GOV, 94. 10.2172/1866610

[B11] Mosier N. Wyman C. Dale B. Elander R. Lee Y. Y. Holtzapple M. (2005). Features of promising technologies for pretreatment of lignocellulosic biomass. Bioresour. Technol. 96 (6), 673–686. 10.1016/j.biortech.2004.06.025 15588770

[B12] Qiao H. Wang Y. Ma Z. Han M. Zheng Z. Ouyang J. (2023). In-depth investigation of formic acid pretreatment for various biomasses: chemical properties, structural features, and enzymatic hydrolysis. Bioresour. Technol. 374, 128747. 10.1016/j.biortech.2023.128747 36804857

[B13] Rabelo S. C. Nakasu P. Y. S. Scopel E. Araújo M. F. Cardoso L. H. Costa A. C. D. (2023). Organosolv pretreatment for biorefineries: current status, perspectives, and challenges. Bioresour. Technol. 369, 128331. 10.1016/j.biortech.2022.128331 36403910

[B14] Saha B. C. Iten L. B. Cotta M. A. Wu Y. V. (2005). Dilute acid pretreatment, enzymatic saccharification and fermentation of wheat straw to ethanol. Process Biochem. 40 (12), 3693–3700. 10.1016/j.procbio.2005.04.006 15932261

[B15] Sluiter A. Hames B. Ruiz R. Scarlata C. Sluiter J. Templeton D. (2012). Determination of Structural Carbohydrates and Lignin in Biomass. Golden, Colorado, USA: National Renewable Energy Laboratory NREL. Available online at: https://www.nrel.gov/bioenergy/biomass-compositional-analysis.html (Accessed April 22, 2023).

[B16] Werpy T. Peterson G. (2004). Top Value Added Chemicals from Biomass: Volume I--Results of Screening for Potential Candidates from Sugars and Synthesis Gas. 10.2172/15008859

[B17] Zhang C. Lei X. Scott C. T. Zhu J. Li K. (2014). Comparison of dilute acid and sulfite pretreatment for enzymatic saccharification of earlywood and latewood of douglas fir. Bioenerg. Res. 7, 362–370. 10.1007/s12155-013-9376-6

[B18] Zhu J. Y. Pan X. J. (2010). Woody biomass pretreatment for cellulosic ethanol production: technology and energy consumption evaluation. Bioresour. Technol. 101 (13), 4992–5002. 10.1016/j.biortech.2009.11.007 19969450

