# Supplementary material for: Effects of chemical preconditioning on organic acid production from woody biomass
Source: Front Chem. 2026 Jul 21;14:1879689. doi: 10.3389/fchem.2026.1879689 (PMC13433724; doi:10.3389/fchem.2026.1879689)
Supplement: Supplementary file 1 [file DataSheet1.pdf]

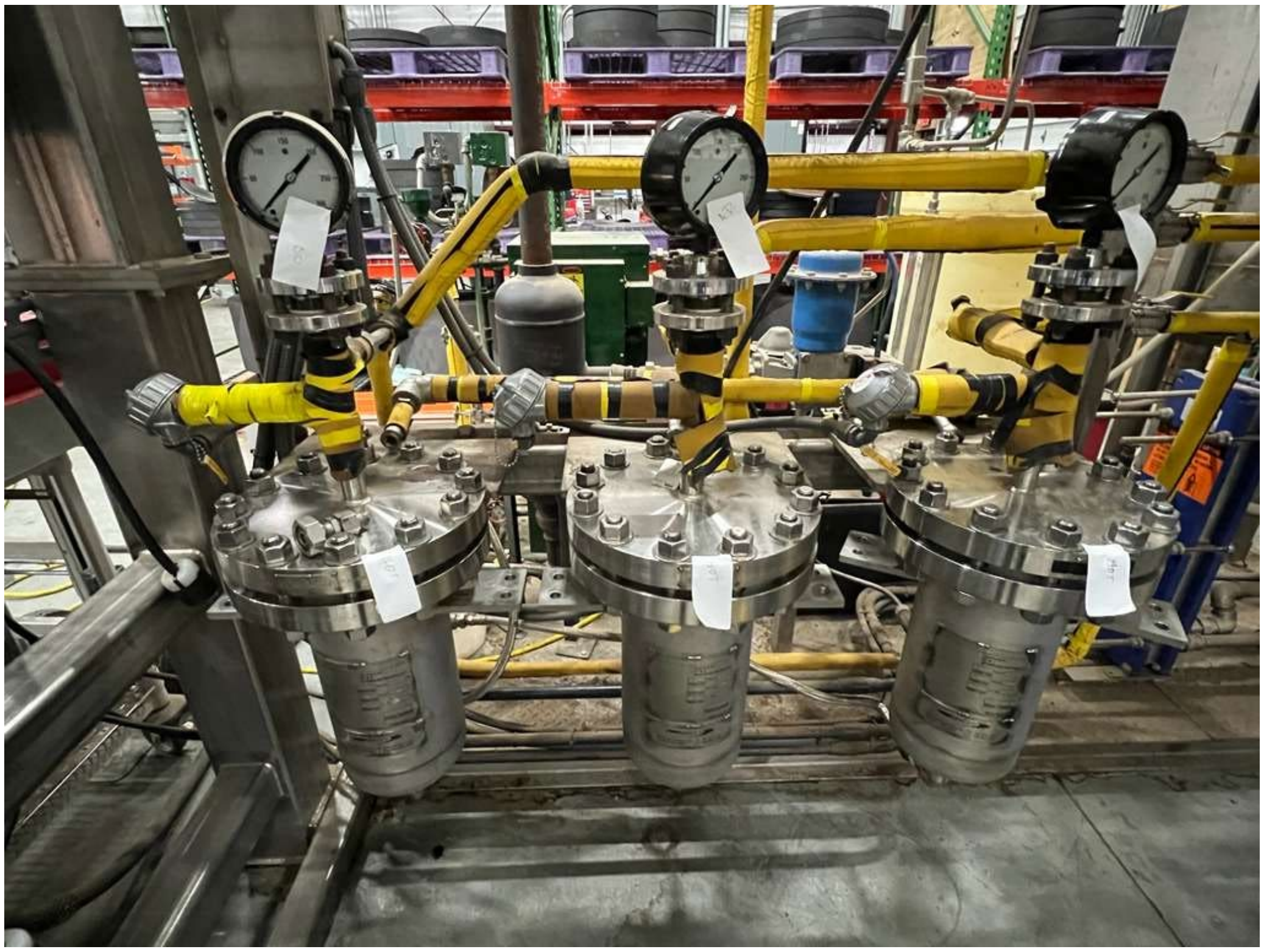

Figure S1: Batch Chemical Preconditioning System (CPS) reactors

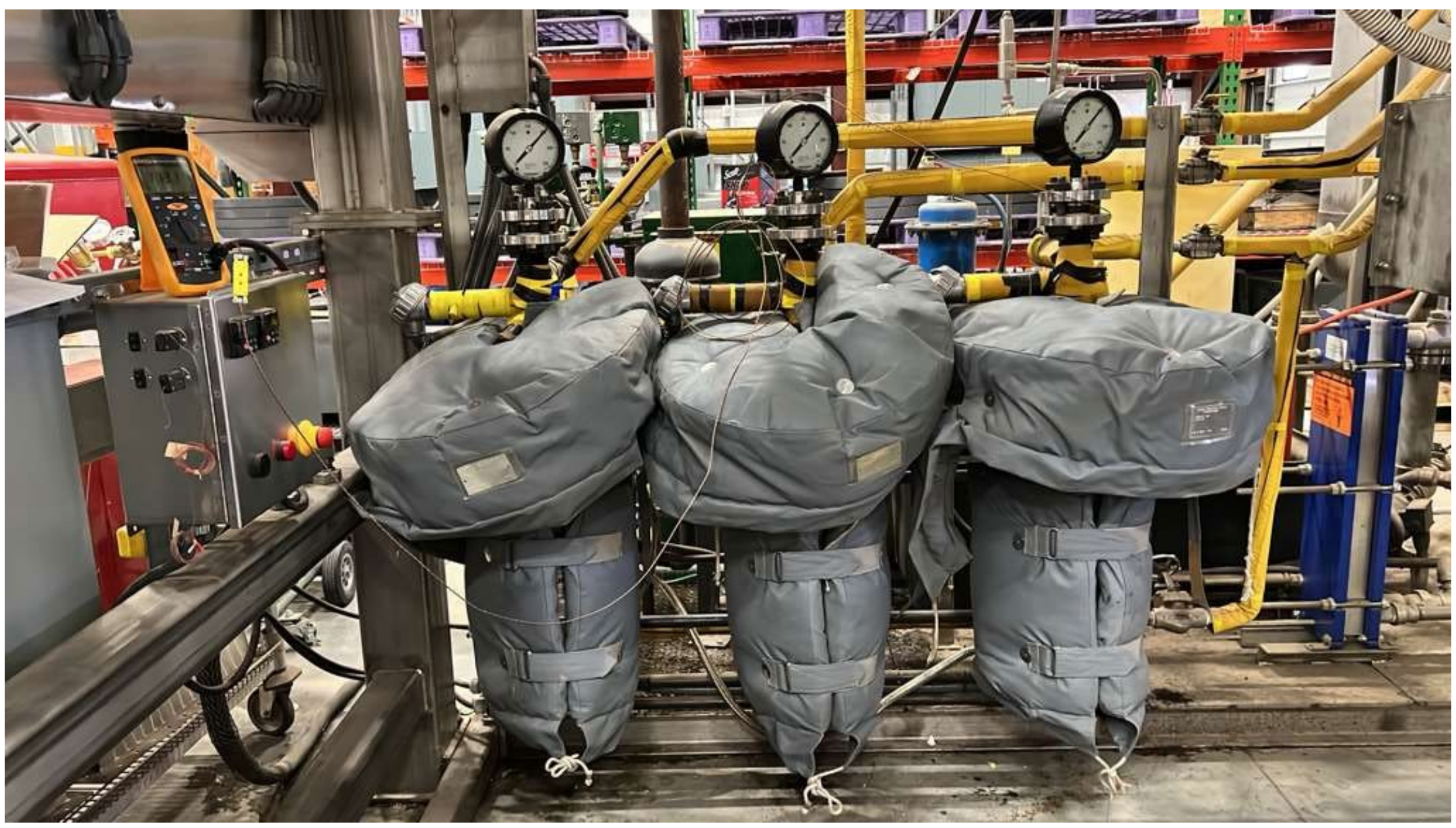

Figure S2: Batch Chemical Preconditioning System (CPS) reactors with heat insulation

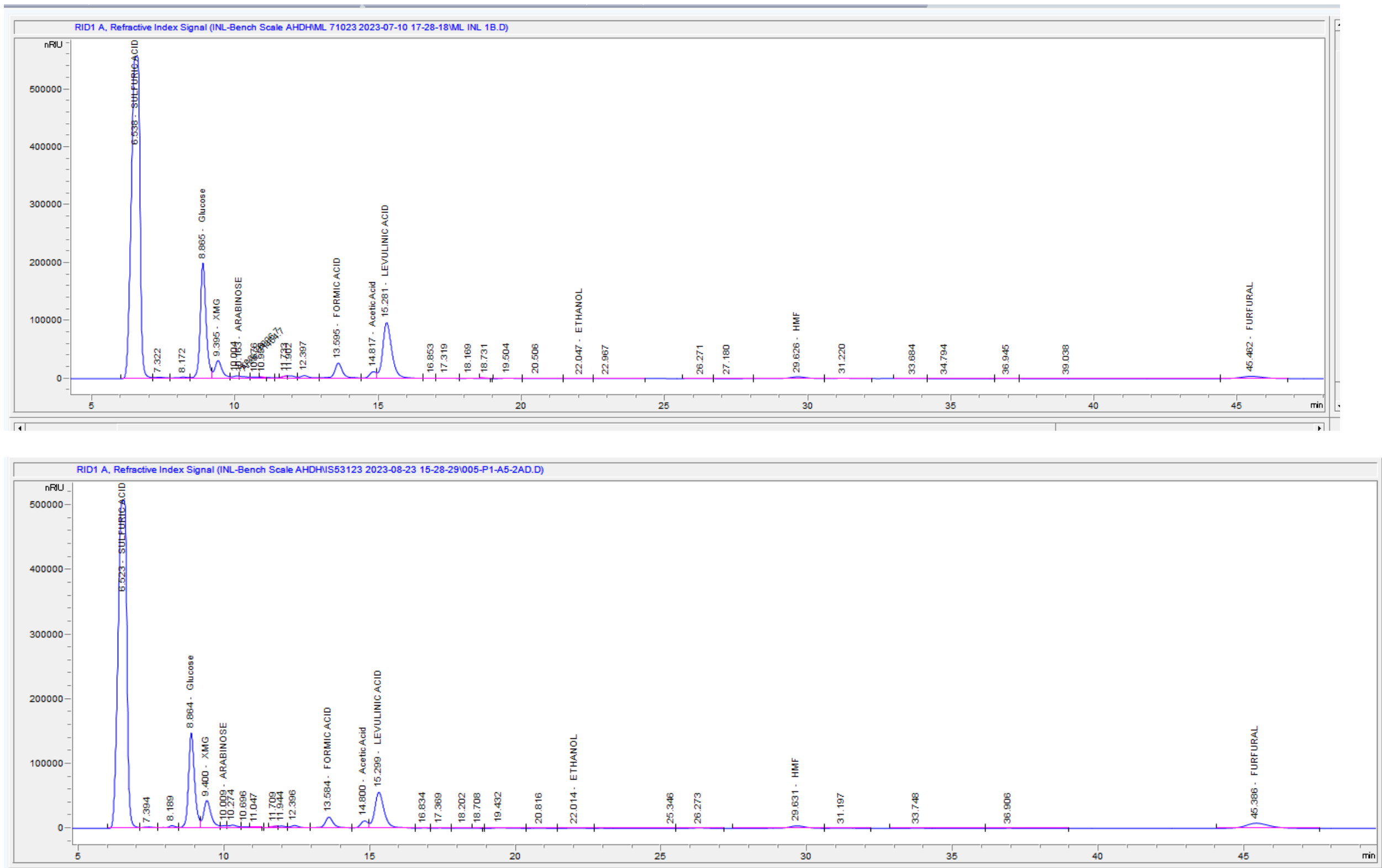

Figure S3: Representative HPLC chromatogram of levulinic acid production from CPS-pretreated solids corresponding to the data in Table 3.
